# Reliability of disc diffusion testing and molecular epidemiology of penicillin-susceptible *Staphylococcus aureus* bacteraemia

**DOI:** 10.1093/jac/dkaf187

**Published:** 2025-06-10

**Authors:** Pernilla Kihlberg, Thor Bech Johannesen, Marc Stegger, Sara Cajander, Bo Söderquist

**Affiliations:** Department of Infectious Diseases, Faculty of Medicine and Health, Örebro University, Örebro, Sweden; Department for Sequencing and Bioinformatics, Statens Serum Institut, Artillerivej 5, Copenhagen 2300, Denmark; European Society of Clinical Microbiology and Infectious Diseases (ESCMID) Study Group for Staphylococci and Staphylococcal Diseases (ESGS), Basel, Switzerland; Department for Sequencing and Bioinformatics, Statens Serum Institut, Artillerivej 5, Copenhagen 2300, Denmark; European Society of Clinical Microbiology and Infectious Diseases (ESCMID) Study Group for Staphylococci and Staphylococcal Diseases (ESGS), Basel, Switzerland; Antimicrobial Resistance and Infectious Diseases Laboratory, Harry Butler Institute, Murdoch University, 90 South Street, Murdoch, Perth, WA 6150, Australia; School of Medical Sciences, Faculty of Medicine and Health, Örebro University, Örebro, Sweden; Department of Infectious Diseases, Faculty of Medicine and Health, Örebro University, Örebro, Sweden; European Society of Clinical Microbiology and Infectious Diseases (ESCMID) Study Group for Staphylococci and Staphylococcal Diseases (ESGS), Basel, Switzerland; School of Medical Sciences, Faculty of Medicine and Health, Örebro University, Örebro, Sweden; Department of Laboratory Medicine, Faculty of Medicine and Health, Örebro University, Örebro SE-701 82, Sweden

## Abstract

**Background:**

Recent studies have reported an increasing prevalence of penicillin-susceptible *Staphylococcus aureus* (PSSA) worldwide. The reliability of disc diffusion testing for detecting penicillin-resistance has been questioned, and the molecular epidemiology of PSSA has not been studied to the same extent as that of MRSA strains.

**Objectives:**

To investigate the reliability of the disc diffusion method for detecting penicillin-resistance in *S. aureus*, and to examine the prevalence and molecular epidemiology of PSSA in bloodstream infections.

**Methods:**

A total of 258 bacteraemic isolates obtained from one geographic region in Sweden during 2018–2019 were analysed using the disc diffusion test to detect penicillin-resistance, and genome sequenced to examine the prevalence of the *blaZ* gene and the molecular epidemiology of PSSA.

**Results:**

Phenotypic susceptibility to penicillin correlated strongly with the absence of the *blaZ* gene, with nearly 98% concordance. The prevalence of PSSA among patients with bacteraemia was 35.1%, highlighting the need for penicillin-susceptibility testing. Additionally, population structure analyses revealed substantial genetic diversity, underscoring the complexity of the PSSA epidemiology. The PSSA belonged to diverse clonal lineages, with CC5 and CC45 dominating our cohort, similar to findings in Spain, Australia, and other parts of Sweden. However, our study revealed a higher prevalence of CC12 compared with other regions, underscoring the importance of local epidemiological surveillance.

**Conclusions:**

These findings validate the reliability of EUCAST’s disc diffusion method, showing a high prevalence of PSSA, and provide insight into the genetic underpinnings of penicillin-susceptibility in *S. aureus.*

## Introduction

*Staphylococcus aureus* bacteraemia (SAB) is a common and life-threatening condition with significant morbidity and a 30-day mortality rate of approximately 20%.^1^ Prior to the introduction of penicillin G in the 1940s, SAB was associated with a mortality rate as high as 82%.^2–4^ The development of penicillin has revolutionized treatment outcomes and substantially reduced mortality.^4^ However, resistance to penicillin emerged shortly after its introduction.^5,6^ By the 1970s, 80%–95% of all SAB strains were resistant to penicillin G,^7^ leading many microbiology laboratories to discontinue susceptibility testing for penicillin in *S. aureus.*^8^

Penicillin G, a β-lactam antibiotic, inhibits penicillin-binding proteins that are essential for bacterial cell wall synthesis, ultimately causing cell death.^9^ Resistance to penicillin in *S. aureus* is mediated by the *blaZ* gene, which encodes penicillinase, an enzyme that hydrolyses the antibiotic’s β-lactam ring and thus renders it inactive.^10^ The *blaZ* gene is regulated by the *blaR1* and *blaI* genes, and four types (A–D) have been described in *S. aureus*, located either on plasmids or on the chromosome.^11–13^ To counteract this resistance, semi-synthetic penicillinase-stable antibiotics such as oxacillin and flucloxacillin have been developed and are widely used for treating *S. aureus* infections.

Despite the historical dominance of penicillin-resistant *S. aureus* (PRSA), recent studies indicate an increasing prevalence of penicillin-susceptible *S. aureus* (PSSA), with rates ranging from 9% to 30% globally, depending on the region.^8,14–18^ This resurgence of PSSA has sparked renewed interest in penicillin G as a treatment option, given its favourable pharmacokinetic profile, including lower toxicity and reduced protein binding compared with isoxazolyl-penicillins.^8,19^ The optimal treatment approach for PSSA remains unclear, but retrospective studies have suggested superior outcomes when SAB is treated with penicillin G compared with cloxacillin or flucloxacillin.^20,21^

The reliability of phenotypic methods such as the disc diffusion test in detecting penicillin-susceptibility is a critical factor in the clinical decision-making process. The CLSI and EUCAST guidelines recommend disc diffusion testing using 1 and 10 U penicillin discs, respectively (Figure S1, available as Supplementary data at *JAC* Online).^19,22^ Although the test is generally reliable, variability in interpretation has been reported, particularly in zone edge assessment.^23^ Further complicating matters, the American Heart Association discourages the use of penicillin G for endocarditis caused by PSSA due to concerns about testing accuracy.^24^

The molecular epidemiology of emerging MRSA strains has been extensively studied, revealing the propagation of several clonal lineages with distinct genetic backgrounds.^25^ In contrast, our understanding of PSSA epidemiology is much more limited. However, previous studies have shown that the emergence of PSSA is not due to expansion of one nor a few dominant clones.^14,17,26,27^

This study aimed to evaluate the correlation between phenotypic and genotypic resistance of PSSA, to assess the reliability of EUCAST’s disc diffusion method, and to investigate the molecular epidemiology, genetic resistance markers, and routes of acquisition for PSSA in SAB.

## Materials and methods

### Ethics

Ethical approval was obtained from the regional ethical board in Uppsala, Sweden (reference number: 2019-04415), and the study was conducted in accordance with the 1964 Helsinki Declaration and its later amendments. The requirement for informed consent was waived due to the retrospective nature of this study.

### Bacterial isolates and patient data

This population-based study was conducted in Örebro County, Sweden, from 2018 to 2019, covering a municipality of approximately 300 000 inhabitants. Clinical isolates were collected from adult patients (≥18 years) with confirmed SAB (defined as *S. aureus* present in one or more blood cultures) and analysed at the Department of Laboratory Medicine, Clinical Microbiology, Örebro University Hospital. The isolates were stored in a preservation broth [trypticase soy broth with 0.3% (w/w) yeast extract and 29% (v/w) horse serum] at −80°C. Exclusion criteria included polymicrobial bacteraemia (unless assessed as contamination according to medical charts), incomplete data, or special confidentiality restrictions in medical records. Patient charts were reviewed to confirm eligibility and to classify the route of acquisition as community-acquired, healthcare-associated, or nosocomial.^28^

### Penicillin-susceptibility testing

**Figure 1.**
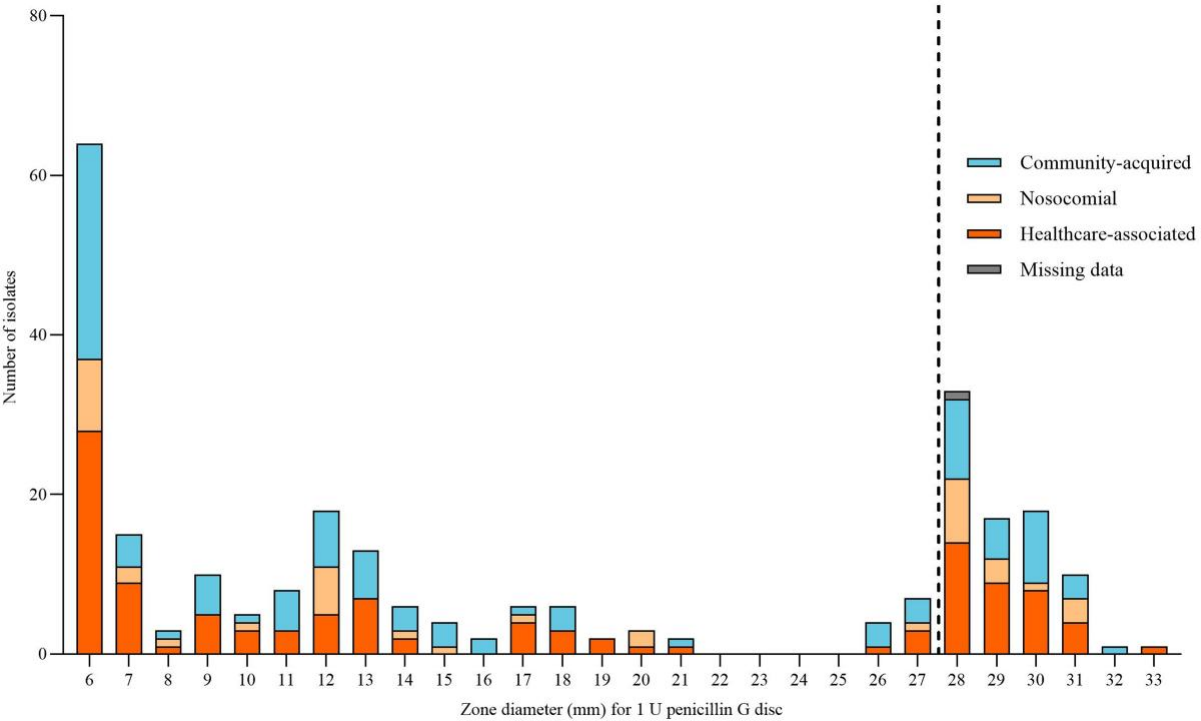
Disc diffusion test for 258 *Staphylococcus aureus* isolates with 1 U penicillin G disc according to the EUCAST guidelines. Mean zone diameter of two investigators. Strains with zone diameters of 6–21 mm were penicillin-resistant, and strains with zone diameters of 26–33 mm were penicillin-susceptible, as marked by the dotted line.

Disc diffusion tests were performed according to EUCAST guidelines.^19^ A standardized inoculum was prepared by suspending bacterial colonies in sterile saline to achieve a turbidity equivalent to a 0.5 McFarland standard, and inoculated on Mueller-Hinton agar plates using 1 U penicillin G discs (Oxoid, Thermo Fisher Scientific, Basingstoke, UK). The plates were incubated in air at 35 ± 1°C for 18 ± 2 h. Zone diameters and edge appearances were assessed by two independent investigators (Figure 1). *S. aureus* reference isolates ATCC 29213 and ATCC 25923 were included as quality controls.

### Whole-genome sequencing

Genomic DNA was extracted on a MagNA Pure 96 automated extraction platform (Roche, Basel, Switzerland) using the Viral NA Small Volume DNA Multi-Sample Kit (Roche), all according to manufacturers’ instructions. Quant-iT dsDNA BR and HS Assay kits (Thermo Fisher Scientific, Carlsbad, CA, USA) were used for DNA quantification and measured on the FLUOstar Omega (BMG LabTech). Libraries were prepared using the Illumina Nextera XT DNA Library Preparation Kit (Illumina, San Diego, CA, USA) and sequenced on the NextSeq 550 platform (Illumina) using a 300-cycle kit for paired-end 150 bp reads.

### Genomic assembly and phylogenetic reconstruction

Draft genomes were assembled using version 3.13.1 of SPAdes.^29^ From these, *blaZ* alleles were identified using BLASTN and parsed for nonsense mutations to identify incomplete genes. *In silico* multilocus sequence typing was performed using mlst (https://github.com/tseemann/mlst) on the assembled genomes.

A core genome single nucleotide polymorphism (SNP) alignment was generated using version 1.2 of the Northern Arizona SNP Pipeline,^30^ with the chromosome of *S. aureus* NC_021554 as reference. This was subsequently used to infer phylogenetic relatedness using version 2.3.0 of IQ-TREE^31^ based on an alignment of 146 537 SNPs in a core genome of 2.0 Mbp (70%). The phylogenetic tree was visualized and annotated using iTOL (https://itol.embl.de/).

### Statistics

Proportions were compared using Pearson’s chi-square test in version 29 of IBM SPSS Statistics. *P*-values <0.05 were considered statistically significant.

## Results

### Patient flow

In total, 296 *S. aureus* strains were identified, of which 25 were excluded due to polymicrobial bacteraemia, two were excluded due to incomplete data in medical charts, and seven were excluded due to confidentiality restrictions in medical records (Figure S2). Additionally, one strain was not subjected to Whole-genome sequencing (WGS), and one strain was not available for phenotypically testing due to technical issues. Two strains were identified as methicillin-resistant and were excluded from the analyses. This resulted in a final total of 258 *S. aureus* strains included for detailed analyses.

### Phenotypic test results

**Figure 2.**
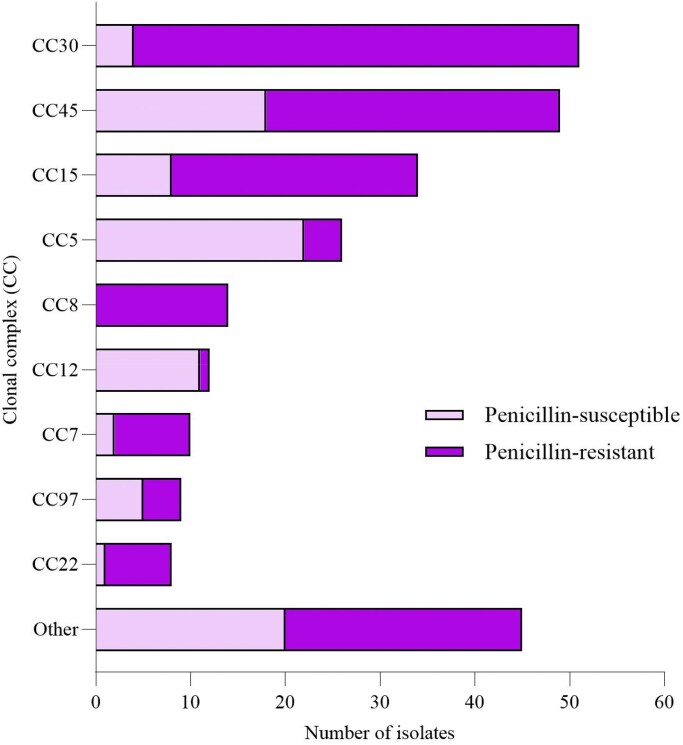
Proportions of CCs of penicillin-resistant and penicillin-susceptible *Staphylococcus aureus* strains.

**Figure 3.**
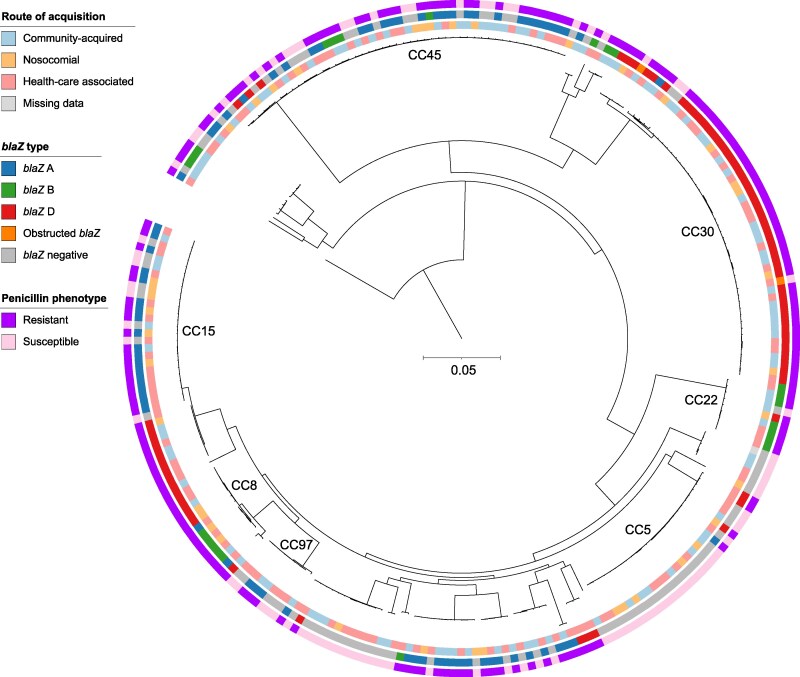
A phylogenetic tree based on approximately 147 000 single nucleotide polymorphisms (SNPs) among 258 *Staphylococcus aureus* isolates causing bacteraemia. The tree depicts the population structure and highlights the major CCs. Three concentric annotation rings illustrate additional colour-coded metadata for each isolate: Inner ring: Route of acquisition classified as community-acquired, nosocomial or healthcare-associated. Middle ring: Prevalence and type of the *blaZ* gene. Outer ring: Phenotypical susceptibility demonstrating penicillin-resistance or penicillin-susceptibility. The scale bar represents 0.05 nucleotide substitutions per site.

Of the included isolates, 91 (35.1%) were phenotypically classified as PSSA (Figures 1–3). The assessments by the two independent investigators were 100% congruent when the zone diameter and zone edge appearance were evaluated together. Among these isolates, 89/91 (97.8%) did not carry the *blaZ* gene. Two (2.2%) phenotypically PSSA isolates, both belonging to clonal complex (CC) 30, were found to harbour the *blaZ* gene type D (Figure 3). However, one of these isolates lacked the usual start codon of the gene, while the other had a frameshift mutation caused by a single nucleotide insertion within the first 10 nucleotides of *blaZ.*

The phenotypically PRSA isolates (*n* = 167) all harboured the *blaZ* gene. All four *blaZ* types were represented among these isolates: type A (*n* = 72; 43.1%), type B (*n* = 26; 15.6%), type C (*n* = 68; 40.7%), and type D (*n* = 1; 0.6%) (Figure 3).

### Population structure

The population structure of the PSSA isolates (*n* = 91) was heterogeneous, with isolates distributed across multiple clonal complexes: CC5 (*n* = 22; 24.2%), CC45 (*n* = 18; 19.8%), CC12 (*n* = 11; 12.1%), CC15 (*n* = 8; 8.8%), CC97 (*n* = 5; 5.5%), CC30 (*n* = 4; 4.4%), CC7 (*n* = 2; 2.2%), CC22 (*n* = 1; 1.1%), and other (*n* = 20; 22.0%) (Figures 2 and 3).

### Route of acquisition

The SAB was community-acquired in 103 (39.8%) cases, healthcare-associated in 114 (44.0%) cases, and nosocomial in 40 (15.4%) cases (Figures 1 and 3). In one case, the route of acquisition could not be determined due to insufficient information in the medical records. There was no statistically significant difference in the route of transmission between PRSA and PSSA (*P* = 0.73).

## Discussion

The disc diffusion test according to EUCAST guidelines demonstrated high reliability in detecting PSSA by accurately differentiating strains with functional *blaZ* genes from those lacking or harbouring non-functional variants. In this study, nearly 98% of the isolates showed concordance between phenotypic resistance and *blaZ* status, emphasising the test’s robustness. Notably, 2.2% of PSSA isolates carried non-functional *blaZ* genes (type D). This is consistent with previous findings by Mama *et al*.^26^ who reported a 3.2% *blaZ-*positivity rate among PSSA isolates in Spain with various mutations in the *blaZ* gene (types A, B, and C). Jin *et al*.^17^ reported a slightly higher prevalence of *blaZ*-positive PSSA (7.9%) in China (types A and C). These findings, obtained through WGS, also revealed notable mutations in the *blaZ* gene and its regulatory gene *blaR1*. Interestingly, Coombs *et al*.^27^ found 9.6% *blaZ*-positive PSSA in Australia in 2020. Here, PSSA was defined according to results obtained from the automated microbiology system Vitek2, and when confirming the susceptibility with the disc diffusion test, all *blaZ*-positive strains were found to be phenotypically PRSA.

Our study identified a relatively high prevalence (35.1%) of PSSA among bacteraemic patients. This is consistent with the 33.1% reported by Resman *et al*.^14^ from southern Sweden, but stands in contrast to the lower prevalence rates reported from Spain (20.7%)^26^ and Australia (19.4%).^27^ Encouraging upward trends in PSSA prevalence have been observed both in Sweden between 1980 and 2010^32^ and in China between 2014 (3.5%) and 2019 (22.1%).^17^ The re-emergence of PSSA in recent years is not fully understood but is most likely driven by a combination of several factors. Resistance is often associated with a fitness cost for the bacteria, and a reduction in the selective pressure with the diminishing use of penicillin in favour of broad-spectrum antimicrobials might have given penicillin-susceptible strains a competitive advantage.^33,34^

The molecular epidemiology of PSSA in our cohort showed that CC5 and CC45 were the most prevalent CCs. These findings align with data from earlier studies in Sweden, Spain, and Australia.^14,26,27^ However, comparisons also highlight regional differences. For instance, CC398 was common in China and Spain, but was absent in our cohort as well as in an earlier Swedish study, and CC12 was more prevalent in our study than in previous reports.^14,17,26,27^ These findings highlight both shared trends and regional variations in the CC distribution.

We found no significant association between penicillin-resistance and the route of acquisition, with PSSA and PRSA being evenly distributed among community-acquired, healthcare-associated, and nosocomial infections. To the best of our knowledge, this aspect has not been previously investigated, although healthcare-associated and nosocomial infections are often associated with multidrug resistance.^35^

The interpretation of disc diffusion tests remains a critical component of PSSA detection. While our study achieved 100% concordance between investigators in the combined assessment of zone diameter and edge appearance, previous studies have highlighted variability in this respect. Hombach *et al*.^36^ demonstrated inter-individual variability among nine investigators when evaluating the performance of the disc diffusion test in discriminating between PSSA and PRSA. However, the sensitivity was likely related to the training level of the investigator. Moreover, in their study, the gold standard was based on detection of the *blaZ* gene without further investigation of mutations.

The presence of non-functional *blaZ* genes in phenotypically susceptible strains poses a potential risk of misclassification. In our study, two strains carried a non-functional *blaZ* gene yet were phenotypically susceptible. Although the clinical significance of non-functional *blaZ* genes remains unclear, concerns have been raised regarding the potential reversibility of *blaZ* mutations. Mama *et al*.^26^ observed revertants in two *blaZ*-positive, phenotypically susceptible strains. Additionally, Eriksen *et al*.^37^ reported a case where a clinical isolate lost a plasmid-borne *blaZ* gene during subculture, converting from resistant to susceptible.

Despite these concerns, retrospective studies have not demonstrated inferior clinical outcomes associated with penicillin treatment of PSSA. For instance, Mok *et al*.^38^ reported no significant differences in mortality or treatment failure between PSSA patients treated with penicillin and those treated with other agents. Furthermore, previous studies have found that treatment with isoxazolyl-penicillin was associated with higher mortality than treatment with penicillin G.^20,21^ Prospective trials, such as the ongoing *S. aureus* Network Adaptive Platform trial (www.snaptrial.com.au), aim to further elucidate these findings by comparing penicillin with flucloxacillin in bloodstream infections caused by PSSA.^39^ This trial employs disc diffusion testing following EUCAST or CLSI guidelines depending on laboratory practices.^39,40^

### Limitations

This study has several limitations. It was conducted using PSSA isolates obtained from a limited geographical area. Furthermore, only the 1 U penicillin disc was used, as recommended by the EUCAST guidelines, rather than additionally using the 10 U penicillin disc specified in the CSLI guidelines. This might reduce the applicability of the results in regions following CSLI guidelines. However, Skov *et al*.^41^ reported 96% sensitivity and 100% specificity with both 1 U (EUCAST guidelines) and 10 U (CSLI guidelines) penicillin discs when the *blaZ* gene was used as the gold standard. Moreover, Papanicolas *et al*.^42^ found an even greater difference in sensitivity between the 1 U disc (100%) and the 10 U disc (89%).

### Conclusion

In conclusion, the prevalence of PSSA among bacteraemic patients in our study was 35.1%. The disc diffusion test proved reliable for detecting PSSA by discriminating strains with functional *blaZ* genes from those lacking the gene or carrying non-functional variants. Diverse lineages were seen among the PSSA strains, with CC5, CC45, CC12, and CC15 being the most common CCs in our cohort.

## Supplementary Material

dkaf187_Supplementary_Data

## Data Availability

The datasets generated and analysed during the current study are available from the European Nucleotide Archive under BioProject accession number PRJEB85823.

## Supplementary data

Figures S1 and S2 is available as Supplementary data at *JAC* Online.

## References

[1] van Hal SJ, Jensen SO, Vaska VL et al Predictors of mortality in *Staphylococcus aureus* bacteremia. Clin Microbiol Rev 2012; 25: 362–86. 10.1128/CMR.05022-11 22491776 PMC3346297

[2] Ligon BL . Penicillin: its discovery and early development. Semin Pediatr Infect Dis 2004; 15: 52–7. 10.1053/j.spid.2004.02.001 15175995

[3] Skinner D, Keefer CS. Significance of bacteremia caused by *Staphylococcus aureus*. Arch Intern Med 1941; 68: 851–75. 10.1001/archinte.1941.00200110003001

[4] Smith IM, Vickers AB. Natural history of 338 treated and untreated patients with staphylococcal septicaemia (1936-1955). Lancet 1960; 1: 1318–22. 10.1016/S0140-6736(60)92303-5 13831996

[5] Kirby WM . Extraction of a highly potent penicillin inactivator from penicillin resistant staphylococci. Science 1944; 99: 452–3. 10.1126/science.99.2579.452 17798398

[6] Barber M, Rozwadowska-Dowzenko M. Infection by penicillin-resistant staphylococci. Lancet 1948; 2: 641–4. 10.1016/S0140-6736(48)92166-7 18890505

[7] Chambers HF . The changing epidemiology of *Staphylococcus aureus*? Emerg Infect Dis 2001; 7: 178–82. 10.3201/eid0702.010204 11294701 PMC2631711

[8] Cheng MP, René P, Cheng AP et al Back to the future: penicillin-susceptible *Staphylococcus aureus*. Am J Med 2016; 129: 1331–3. 10.1016/j.amjmed.2016.01.048 26924388

[9] Jensen SO, Lyon BR. Genetics of antimicrobial resistance in *Staphylococcus aureus*. Future Microbiol 2009; 4: 565–82. 10.2217/fmb.09.30 19492967

[10] Shalaby MW, Dokla EME, Serya RAT et al Penicillin binding protein 2a: an overview and a medicinal chemistry perspective. Eur J Med Chem 2020; 199: 112312. 10.1016/j.ejmech.2020.112312 32442851

[11] Olsen JE, Christensen H, Aarestrup FM. Diversity and evolution of blaZ from *Staphylococcus aureus* and coagulase-negative staphylococci. J Antimicrob Chemother 2006; 57: 450–60. 10.1093/jac/dki492 16449305

[12] Zhang HZ, Hackbarth CJ, Chansky KM et al A proteolytic transmembrane signaling pathway and resistance to beta-lactams in staphylococci. Science 2001; 291: 1962–5. 10.1126/science.1055144 11239156

[13] Lade H, Kim JS. Molecular determinants of β-lactam resistance in methicillin-resistant *Staphylococcus aureus* (MRSA): an updated review. Antibiotics (Basel) 2023; 12: 1362. 10.3390/antibiotics12091362 37760659 PMC10525618

[14] Resman F, Thegerström J, Månsson F et al The prevalence, population structure and screening test specificity of penicillin-susceptible *Staphylococcus aureus* bacteremia isolates in Malmö, Sweden. J Infect 2016; 73: 129–35. 10.1016/j.jinf.2016.05.011 27265236

[15] Butler-Laporte G, Lee TC, Cheng MP. Increasing rates of penicillin sensitivity in *Staphylococcus aureus*. Antimicrob Agents Chemother 2018; 62: e00680-18. 10.1128/AAC.00680-18 29686148 PMC6021674

[16] Chabot MR, Stefan MS, Friderici J et al Reappearance and treatment of penicillin-susceptible *Staphylococcus aureus* in a tertiary medical centre. J Antimicrob Chemother 2015; 70: 3353–6. 10.1093/jac/dkv270 26342027

[17] Jin Y, Zhou W, Zhan Q et al Genomic epidemiology and characterisation of penicillin-sensitive *Staphylococcus aureus* isolates from invasive bloodstream infections in China: an increasing prevalence and higher diversity in genetic typing be revealed. Emerg Microbes Infect 2022; 11: 326–36. 10.1080/22221751.2022.2027218 34991434 PMC8786255

[18] Richter SS, Doern GV, Heilmann KP et al Detection and prevalence of penicillin-susceptible *Staphylococcus aureus* in the United States in 2013. J Clin Microbiol 2016; 54: 812–4. 10.1128/JCM.03109-15 26763960 PMC4767943

[19] EUCAST . Clinical breakpoints—bacteria (v 12.0). https://www.eucast.org/clinical_breakpoints/.

[20] Henderson A, Harris P, Hartel G et al Benzylpenicillin versus flucloxacillin for penicillin-susceptible *Staphylococcus aureus* bloodstream infections from a large retrospective cohort study. Int J Antimicrob Agents 2019; 54: 491–5. 10.1016/j.ijantimicag.2019.05.020 31181352

[21] Hagstrand Aldman M, Kavyani R, Kahn F et al Treatment outcome with penicillin G or cloxacillin in penicillin-susceptible *Staphylococcus aureus* bacteraemia: a retrospective cohort study. Int J Antimicrob Agents 2022; 59: 106567. 10.1016/j.ijantimicag.2022.106567 35288257

[22] CLSI . *Performance Standards for Antimicrobial Susceptibility Testing—Thirty-Third Edition: M100*. 2023.

[23] El Feghaly RE, Stamm JE, Fritz SA et al Presence of the bla(Z) beta-lactamase gene in isolates of *Staphylococcus aureus* that appear penicillin susceptible by conventional phenotypic methods. Diagn Microbiol Infect Dis 2012; 74: 388–93. 10.1016/j.diagmicrobio.2012.07.013 22959917

[24] Baddour LM, Wilson WR, Bayer AS et al Infective endocarditis in adults: diagnosis, antimicrobial therapy, and management of complications: a scientific statement for healthcare professionals from the American Heart Association. Circulation 2015; 132: 1435–86. 10.1161/CIR.0000000000000296 26373316

[25] Lakhundi S, Zhang K. Methicillin-resistant Staphylococcus aureus: molecular characterization, evolution, and epidemiology. Clin Microbiol Rev 2018; 31: e00020-18. 10.1128/CMR.00020-18 30209034 PMC6148192

[26] Mama OM, Aspiroz C, Lozano C et al Penicillin susceptibility among invasive MSSA infections: a multicentre study in 16 Spanish hospitals. J Antimicrob Chemother 2021; 76: 2519–27. 10.1093/jac/dkab208 34245259

[27] Coombs GW, Yee NWT, Daley D et al Molecular epidemiology of penicillin-susceptible *Staphylococcus aureus* bacteremia in Australia and reliability of diagnostic phenotypic susceptibility methods to detect penicillin susceptibility. Microorganisms 2022; 10: 1650. 10.3390/microorganisms10081650 36014068 PMC9413241

[28] Friedman ND, Kaye KS, Stout JE et al Health care–associated bloodstream infections in adults: a reason to change the accepted definition of community-acquired infections. Ann Intern Med 2002; 137: 791–7. 10.7326/0003-4819-137-10-200211190-00007 12435215

[29] Bankevich A, Nurk S, Antipov D et al SPAdes: a new genome assembly algorithm and its applications to single-cell sequencing. J Comput Biol 2012; 19: 455–77. 10.1089/cmb.2012.0021 22506599 PMC3342519

[30] Sahl JW, Lemmer D, Travis J et al NASP: an accurate, rapid method for the identification of SNPs in WGS datasets that supports flexible input and output formats. Microb Genom 2016; 2: e000074. 10.1099/mgen.0.000074 28348869 PMC5320593

[31] Minh BQ, Schmidt HA, Chernomor O et al Corrigendum to: IQ-TREE 2: new models and efficient methods for phylogenetic inference in the genomic era. Mol Biol Evol 2020; 37: 2461. 10.1093/molbev/msaa131 32556291 PMC7403609

[32] Rasmussen G, Monecke S, Brus O et al Long term molecular epidemiology of methicillin-susceptible *Staphylococcus aureus* bacteremia isolates in Sweden. PLoS One 2014; 9: e114276. 10.1371/journal.pone.0114276 25479442 PMC4257557

[33] Andersson DI, Hughes D. Antibiotic resistance and its cost: is it possible to reverse resistance? Nat Rev Microbiol 2010; 8: 260–71. 10.1038/nrmicro2319 20208551

[34] Jia K, Zhu H, Wang J et al Fitness cost and compensatory evolution of penicillin-induced resistant *Staphylococcus aureus*. Food Res Int 2025; 203: 115841. 10.1016/j.foodres.2025.115841 40022365

[35] US Centers for Disease Control and Prevention. Antimicrobial resistance threats in the United States, 2021–2022. 2024. https://www.cdc.gov/antimicrobial-resistance/dataresearch/threats/update-2022.html.

[36] Hombach M, Weissert C, Senn MM et al Comparison of phenotypic methods for the detection of penicillinase in *Staphylococcus aureus* and proposal of a practical diagnostic approach. J Antimicrob Chemother 2017; 72: 1089–93. 10.1093/jac/dkw521 28069883

[37] Eriksen HB, Petersen A, Pedersen M et al Possible misinterpretation of penicillin susceptibility in *Staphylococcus aureus* blood isolate due to in vitro loss of the blaZ gene. Eur J Clin Microbiol Infect Dis 2022; 41: 163–7. 10.1007/s10096-021-04344-w 34529167

[38] Mok HT, Teng CB, Bergin S et al Treatment outcomes with benzylpenicillin and non-benzylpenicillin antibiotics, and the performance of the penicillin zone-edge test versus molecular detection of blaZ in penicillin-susceptible *Staphylococcus aureus* (PSSA) bacteraemia. J Antimicrob Chemother 2023; 78: 2515–23. 10.1093/jac/dkad263 37596905

[39] Tong SYC, Mora J, Bowen AC et al The *Staphylococcus aureus* network adaptive platform trial protocol: new tools for an old foe. Clin Infect Dis 2022; 75: 2027–34. 10.1093/cid/ciac476 35717634 PMC9710697

[40] Henderson A, Cheng MP, Chew KL et al A multi-site, international laboratory study to assess the performance of penicillin susceptibility testing of *Staphylococcus aureus*. J Antimicrob Chemother 2023; 78: 1499–504. 10.1093/jac/dkad116 37071589 PMC10232234

[41] Skov R, Lonsway DR, Larsen J et al Evaluation of methods for detection of β-lactamase production in MSSA. J Antimicrob Chemother 2021; 76: 1487–94. 10.1093/jac/dkab032 33615356 PMC9479532

[42] Papanicolas LE, Bell JM, Bastian I. Performance of phenotypic tests for detection of penicillinase in *Staphylococcus aureus* isolates from Australia. J Clin Microbiol 2014; 52: 1136–8. 10.1128/JCM.03068-13 24452169 PMC3993472

