# Supplementary material for: Reliability of disc diffusion testing and molecular epidemiology of penicillin-susceptible Staphylococcus aureus bacteraemia
Source: J Antimicrob Chemother. 2025 Jun 10;80(8):2187–93. doi: 10.1093/jac/dkaf187 (PMC12313465; doi:10.1093/jac/dkaf187)
Supplement: dkaf187_Supplementary_Data [file dkaf187_supplementary_data.docx]

**Supplementary**


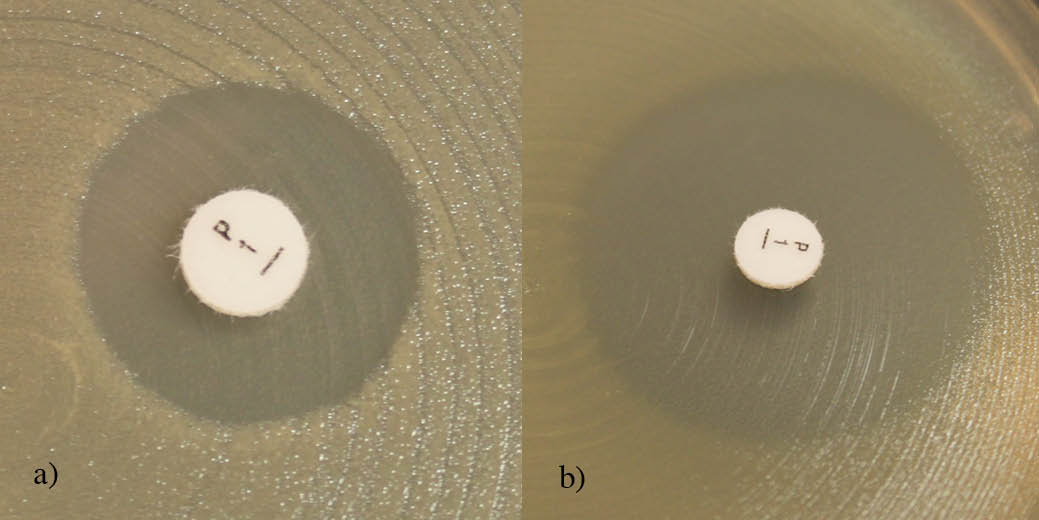


Figure S1. Disc diffusion test for penicillin susceptibility, showing (a) sharp and (b) fuzzy zone edges.


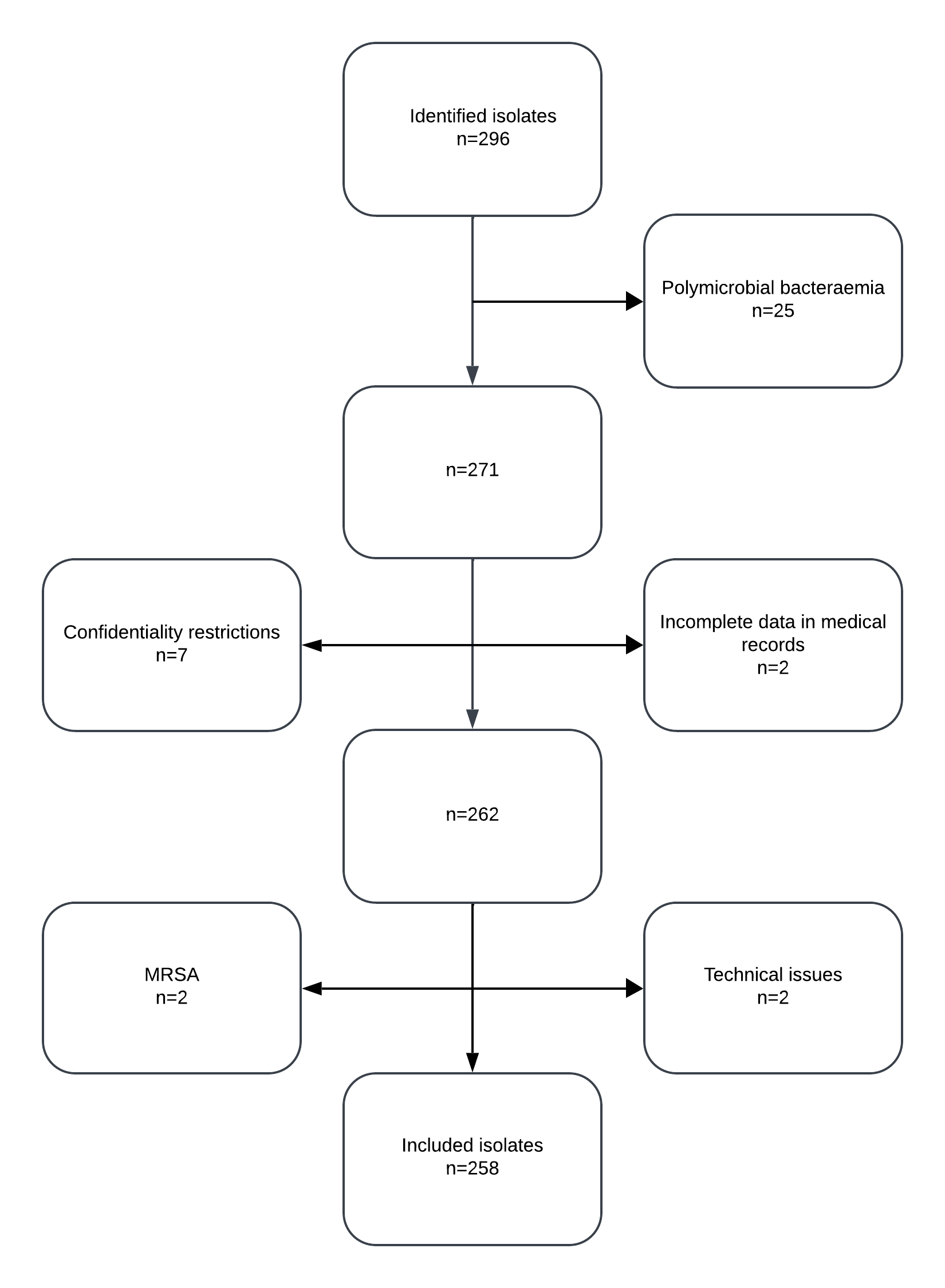


Figure S2. Flowchart showing how isolates from patients with *Staphylococcus aureus* bacteraemia were selected for the present study.
